# Accessibility of Referent Information Influences Sentence Planning: An Eye-Tracking Study

**DOI:** 10.3389/fpsyg.2017.00250

**Published:** 2017-02-28

**Authors:** Lesya Y. Ganushchak, Agnieszka E. Konopka, Yiya Chen

**Affiliations:** ^1^Leiden University Centre for Linguistics, Leiden UniversityLeiden, Netherlands; ^2^Education and Child Studies, Faculty of Social and Behavioral Sciences, Leiden UniversityLeiden, Netherlands; ^3^Department of Psychology, Education and Child Studies, Erasmus University RotterdamRotterdam, Netherlands; ^4^School of Psychology, University of AberdeenAberdeen, UK; ^5^Leiden Institute for Brain and Cognition, Leiden UniversityLeiden, Netherlands

**Keywords:** sentence planning, eye-tracking, givenness, incrementality, discourse context, accessibility

## Abstract

This study investigated the time-course of online sentence formulation (i.e., incrementality in sentence planning) as a function of the preceding discourse context. In two eye-tracking experiments, participants described pictures of transitive events (e.g., a frog catching a fly). The accessibility of the agent (Experiment 1) and patient (Experiment 2) was manipulated in the discourse preceding each picture. In the *Literal* condition, participants heard a story where the agent or patient was mentioned explicitly (*fly, frog*). In the *Associative* condition, the agent or patient was not mentioned but was primed by the story (via semantically or associatively related words such as *insect, small, black, wings*). In the *No Mention* condition, the stories did not explicitly mention or prime either character. The target response was expected to have the same structure and content in all conditions (SVO sentences: *The frog catches the fly*). The results showed that participants generally looked first at the agent, before speech onset, regardless of condition, and then at the patient around and after speech onset. Analyses of eye movements in time window associated with linguistic planning showed that formulation was sensitive mainly to whether the agent was literally mentioned in the context or not and to lesser extent to conceptual accessibility (Experiment 1). Furthermore, accessibility of the patient (be it literal mention of its name or only availability of the concept) showed no effect on the time-course of utterance planning (Experiment 2). Together, these results suggest that linguistic planning before speech onset was influenced only by the accessibility of the first character name in the sentence, providing further evidence for highly incremental planning in sentence production.

## Introduction

To produce a sentence, speakers need to prepare a preverbal message and then encode it linguistically. Preparation and encoding of messages and sentences are assumed to proceed incrementally (e.g., Kempen and Hoenkamp, 1987; Konopka and Brown-Schmidt, 2014). Evidence for incremental planning, however, has come mainly from work on production of individual sentences out of context. Despite the communicative function of speech, little is known about planning of utterances as a function of the discourse context in which they are produced. The aim of this project is to investigate how sentence planning is affected by discourse context. In particular, we will examine how *accessibility* of information about characters in simple events (manipulated as specific information provided about these characters in the preceding discourse context) affects the *time-course* of planning.

Accessibility of information about any element of the preverbal message and corresponding utterance is assumed to be a continuous measure of the degree to which a referent is “active” in that particular context. Any referential expression produced in a sentence (e.g., *the frog* or *it*) may be placed on a “continuum” of accessibility, depending on the context. At one end of the continuum, an expression can be completely new, or inactive in the speaker’s mind, and may need to be encoded from scratch, both conceptually and linguistically, during the planning of an utterance. At the other end of the continuum, a referring expression can be regarded as being completely given and active in the speaker’s mind at the time of planning. This variability in the accessibility of a given concept can be driven by multiple factors, such as attention allocation and discourse context. For example, referents can vary in accessibility due to explicit mention or to activation from other sources (such as activation of conceptually related information in the larger discourse or non-linguistic information, like pictures and gestures; e.g., Clark and Marshall, 1981; Baumann and Hadelich, 2003; Kahn and Arnold, 2012). Accessibility of an event character can also be related to the notion of *givenness*.

Within the psycholinguistic tradition, evidence abounds that the *accessibility* of a referent influences production preferences, both in terms of speakers’ choices of syntactic structures and the time-course of sentence formulation. For example, it has been repeatedly shown that speakers have a strong preference to begin sentences with accessible characters than less accessible characters (e.g., MacWhinney and Bates, 1978; Bock and Warren, 1985; Kempen and Hoenkamp, 1987; McDonald et al., 1993; Branigan and Feleki, 1999; Ferreira and Yoshita, 2003; Kempen and Harbusch, 2003; Christianson and Ferreira, 2005; Tanaka et al., 2011; Konopka and Meyer, 2014; Norcliffe et al., 2015; also see Gleitman et al., 2007, and Kuchinsky and Bock, 2010, for a discussion of perceptual accessibility). It is important to note, however, that these studies primarily examined sentence production out of context. Furthermore, accessibility in these studies is typically discussed in terms of the conceptual features of a referent (e.g., animate vs. inanimate referents), conceptual complexity (e.g., abstract vs. concrete concepts), and ease of naming (e.g., high-frequency vs. low-frequency words).

In addition, accessibility has been manipulated experimentally by increasing word activation levels via lexical primes (i.e., words that are semantically or associatively related to the target referents; Bock, 1986; Konopka and Meyer, 2014). The effect of both the inherent accessibility and experimentally manipulated accessibility of a referent on sentence planning is to facilitate and prioritize the encoding of that referent during the planning process: in eye-tracked production experiments, speakers quickly fixate easy-to-name referents, encode them as sentence subjects, and begin sentences with these references in subject position more quickly than sentences with harder-to-name referents (Konopka and Meyer, 2014). Konopka (2014) provided further evidence that referent accessibility can have a similar effect on sentence planning. Participants described pictures of simple events (e.g., a frog catching a fly) with simple SVO sentences after reading a short sentence that provided either a neutral context or a supporting context for the upcoming target event and that mentioned either the agent or patient explicitly. The results showed that, when speakers produced active sentences, prior mention of the agent reduced not only the length of fixations on that referent but also speech onset in the descriptions that participants generated.

Accessibility can also be approached from the perspective of information integration in a discourse context, in particular the question of how existing information guides the processing of incoming information and how incoming information is in turn integrated with the preceding discourse context. Within the linguistic research tradition, various notions of information status have been posited, with two notably important, if not the most important, concepts: focus and givenness (but see Kruijff, 2001, for a review on various notions posited). A referent is “given” in a particular context if it has been linguistically mentioned and therefore constitutes shared knowledge or recoverable information in a specific context (e.g., Krifka, 2007, and Rochemont, 2016, for reviews). In addition, a referent is in “focus” in a discourse context when it provides new and/or alternative information and consequently advances the discourse (see Gussenhoven, 2007 and Krifka, 2007, for reviews).

There is some existing evidence in the literature that the planning of an utterance *can* be affected by the information status of individual referents in the to-be-articulated messages (e.g., whether the referents are new or known/given). For instance, Konopka (2013, 2014) and Ganushchak et al. (2014) showed that *focus* can affect the time-course of utterance planning. Ganushchak et al. (2014) asked participants to describe pictures of two-character transitive events in Dutch and Chinese, while their eye-movements were recorded. The information status of the characters to be encoded was manipulated by presenting wh-questions prior to each picture (e.g., *What is the policeman stopping? Who is stopping the truck?*). The results showed that speakers rapidly directed their gaze preferentially only to the new (i.e., focused) character they needed to encode before speech onset, suggesting a clear difference in the time-course of planning in sentences with focused and unfocused referents.

While Ganushchak et al. (2014) suggests that given and new characters are planned differently, we know little about how *different levels* of character accessibility affect sentence planning. In particular, there are at least two outstanding questions regarding the effect of different levels of accessibility (i.e., the degree to which information about event characters is accessible in a given context) on planning. First, it is not clear how levels of accessibility of a referent might affect sentence planning in a larger discourse context. Second, it is unclear whether different levels of accessibility of characters that are mentioned early vs. late in a sentence might affect planning in different ways.

**FIGURE 1:**
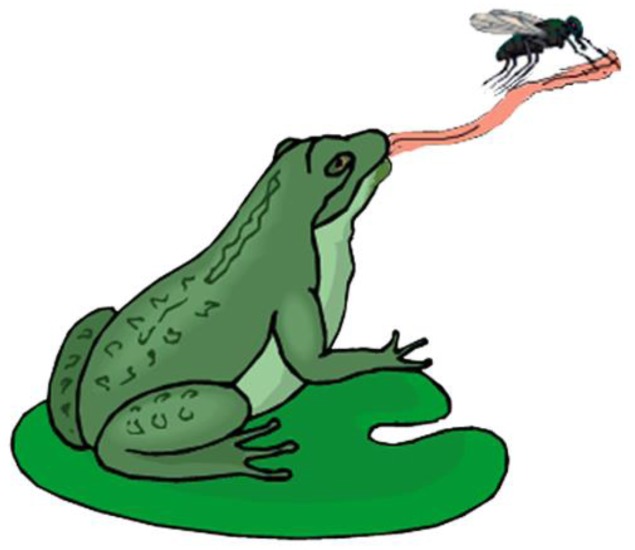
**Example of a target event**.

To address these questions, we compared the time-course of formulation for simple SVO sentences after manipulating the degree of accessibility of the two event characters by simulating a real-life discourse context (**Figure 1** and Examples 1–3). Participants’ eye movements were tracked as they described pictures on a computer screen. The target descriptions were expected to have the same structure and content in all conditions (e.g., *The frog catches the fly* as most events elicited SVO descriptions). The accessibility of the agent (Experiment 1) and patient (Experiment 2) in each event was manipulated in three conditions by means of a short, two-sentence story presented before each picture. In the *No Mention* condition, the stories did not mention or prime either of the event characters. In the *Literal* condition, participants heard a story where either the agent (*frog*) or the patient (*fly*) was explicitly mentioned in a sentence that provided pragmatically appropriate, character-specific information (e.g., *The frog jumped out of the pond*). In the *Associative* condition, the agent or patient was not linguistically mentioned but the story conceptually primed activation of information related to the target character (e.g., the story included words such as *quacking. pond, green*, and *jump*), thereby increasing the accessibility of the target character.

Differences in the planning of the target sentences across conditions were evaluated by comparing speakers’ eye-movements to the agents and patients in the picture prior to speech onset. When preparing to describe such pictures, speakers normally look at characters in the display in the order of mention (Griffin and Bock, 2000). However, there are still debates about the size of planning units during production, i.e., how much of a sentence the speaker typically plans before beginning to speak. Planning units have been shown to vary from units as large as an entire clause to being limited to a single phrase or word (see Konopka, 2012, for a review). Part of this variability can be due to variability in the ease of completing conceptual and linguistic processes required for sentence production.

Broadly speaking, incrementality in sentence planning can be either structural or lexical (Bock et al., 2003, 2004). *Structural* (or *hierarchical*) incrementality posits that sentence formulation begins with the generation of a simple but broad message and sentence plan that captures the relationship between various message elements (i.e., event characters; Bock et al., 2004; Konopka and Meyer, 2014). In some eye-tracking studies, formulation has been shown to begin with a short phase (0–400 ms) during which fixations to the two characters do not differ or do not diverge rapidly (e.g., Griffin and Bock, 2000; Konopka, 2014). This pattern arguably indicates that speakers begin formulation by encoding (or apprehending) the gist of the entire event, suggesting that planning units at the message level can extend well beyond a single concept. Event apprehension is then followed by a longer phase of linguistic encoding during which speakers encode the character names sequentially. During linguistic encoding, easy-to-name characters are typically fixated for less time than harder-to-name characters (e.g., Meyer et al., 1998; Meyer and Lethaus, 2004; Konopka and Meyer, 2014). In contrast, *lexical* (or *linear*) incrementality predicts that a speaker plans the preverbal message one concept at a time and simultaneously plans the linguistic message of the sentence roughly one word at a time (e.g., Gleitman et al., 2007). Indeed, in similar eye-tracked production experiments, speakers have been shown to fixate the subject character very quickly after picture onset (before 400 ms) and the length of fixations on this character also varied with the ease of encoding its name (Gleitman et al., 2007; Kuchinsky, 2009), indicating that, at least under some circumstances, speakers are able to start encoding the preverbal message as well as its linguistic content with one character alone (a very narrow scope of planning). In other words, linear incrementality assumes that speakers can prepare a sequence of small conceptual and linguistic increments without guidance from a higher-level framework.

The current study aims to provide further insight into how character accessibility can affect the scope and time-course of sentence planning. Our first hypothesis concerns eye movements observed immediately after picture onset (0–400 ms, i.e., in a time window associated with a combination of conceptual and linguistic encoding): effects of character accessibility on early eye movements (0–400 ms) should indicate to what extent information in the preceding discourse influences early planning strategies, i.e., the extent to which speakers engage in broad-scope, two-character message planning (hierarchical incrementality) or in narrow-scope, one-character planning (linear incrementality). Early differences in fixations to agents and patients in this window across conditions would indicate variability in *when* speakers begin encoding the agent in SVO sentences. For example, a higher proportion of early fixations to the agent would indicate a shift toward early encoding of this character. This would demonstrate sensitivity to properties of this character and thus sensitivity to context. If the accessibility of the two characters does not influence early formulation, there should then be no difference in gaze patterns across conditions.

A second hypothesis is that accessibility influences planning only at the linguistic encoding (sentence) level. Differences across conditions emerging after 400 ms (i.e., in time windows associated with linguistic encoding) would indicate that accessibility influences primarily *how long* it takes to encode a given character (i.e., the length of linguistic encoding), rather than *when* speakers begin encoding a given character (i.e., a shift in planning strategies). Here, a lower proportion of fixations to the agent as well as shorter fixations to this character indicate shorter lexical retrieval times (e.g., Griffin and Bock, 2000). If character accessibility influences linguistic encoding, then viewing patterns in the *Literal* and *Associative* conditions should differ from the *No Mention* condition: speakers should spend less time fixating the character that was mentioned or that was primed in the story, consistent with the finding that easy-to-name characters are encoded more quickly than harder-to-name characters. Furthermore, fixations on the target character in the *Literal* condition should be shorter than in the *Associative* condition, since the name of this character is ‘given’ in the *Literal* condition but is only temporarily made more ‘accessible’ in the *Associative* condition.

Importantly, predictions from our hypotheses differ for conditions in which the agents versus the patients are “given” in the discourse. In the current experiments, linear incrementality predicts that only the accessibility of the first-mentioned agent (*frog*) should influence gaze patterns because the patient (*fly*) is planned in a separate increment. Thus fixations on the agent in Experiment 1 should vary across conditions, but there should be no differences in early fixations to the two event characters in Experiment 2. In contrast, hierarchical incrementality predicts that participants plan a larger preverbal message (a message that includes information about both the agents and patients) before beginning linguistic encoding, so there may be effects of character accessibility in both experiments. As with manipulations of the accessibility of the agent, fixations to the patient should be shorter in the *Literal* Patient condition than in the *Associate* and *No Mention* conditions in Experiment 2.

## Experiment 1: Accessibility of the Agent

### Method

#### Participants

Thirty native Dutch speakers (23 female) participated in the experiment (mean age: 21.2 years; *SD* = 1.6 years). All participants were students at Dutch universities. The study was approved by the Ethical Committee board at Leiden University. Participants gave written informed consent and received course credit for their participation.

#### Materials

Seventy-eight colored pictures were used in the experiment (Konopka, 2014). All pictures displayed simple actions (**Figure 1**). There were 25 target pictures of transitive events, 50 fillers, and 3 practice pictures. All target pictures had human/animate agents, and thus elicit primarily SVO descriptions. Thirteen out of 25 pictures also had a human/animate patient.

Accessibility was manipulated by means of short stories preceding each picture. All stories consisted of two sentences and were contextually related to the pictures in the three conditions manipulated in the experiment. Take the expected target sentence *De kikker vangt de vlieg* (‘The frog catches the fly’) as an example. The stories presented in the three conditions before picture presentation are listed below:

(1) *No Mention* condition: The story did not mention the words describing the intended target character or include associatively related words.
  *David gaat met zijn vader vissen. Ze gebruiken restjes van het avondeten als aas*. (David is going fishing with his father. They use leftovers from dinner as bait.)
(2) *Literal* condition: The agent was explicitly mentioned in the second sentence of the story and in the same grammatical role as in the intended target sentence.
  *David gaat met zijn vader vissen. Een kikker springt opeens in de sloot*. (David is going fishing with his father. A frog suddenly jumped into the ditch.)
(3) *Associative* condition: The story primed the intended agent. All stories were pre-tested: 20 undergraduate students from Leiden University read all 30 stories one by one and were asked to write down the first word that came to mind after reading each story. The 25 stories with the highest agreement on the intended target word were chosen for the main experiment (agreement > 80%).
  *Koen hoort gekwaak bij de vijver. Als hij gaat kijken, ziet hij iets groens wegspringen*. (Koen heard quacking near the pond. When he went to look, he saw something green jumping away.)

All stories were pre-recorded by a native Dutch female speaker and presented auditorily prior to picture onset in the experiment. On 40% of the filler trials, participants received and answered a yes-or-no comprehension question, presented visually on the computer screen before receiving a picture to describe, to make sure that they listened carefully to the presented stories.

#### Design and Procedure

Lists of stimuli were created to counterbalance story type across target pictures within participants and within items. Each target picture occurred in each condition on a different list so that each participant saw each picture only once. There were at least two filler pictures separating any two target trials in each list.

Participants were seated in a sound-proof room. Eye movements were recorded with an Eyelink 1000 eye-tracker (SR Research Ltd.; 500 Hz sampling rate). A 9-point calibration procedure was performed at the beginning of the experiment. All picture trials began with a fixation point presented at the top of the screen for drift correction. The task started with three practice trials. On all trials, participants first heard a story (presented through headphones). The participants were instructed to listen carefully to the story as sometimes they would be asked a question about it immediately after reading the story. On 40% of the filler items, participants received a yes/no comprehension question and where instructed to use the computer mouse to give their response. The stories were followed by presentation of the pictures that participants had to describe out loud with one sentence, mentioning all the characters in the picture. They were not under time pressure to produce the descriptions and were not instructed to produce sentences with any specific structure. They were also not told about the relationship between the stories and the pictures. When the participant finished speaking, the experimenter clicked with the mouse to proceed to the next trial. On average, the pictures were displayed on the screen for 5227 ms (*SD* = 1604 ms).

#### Scoring and Data Analysis

Trials in which the first fixation was within the agent or patient interest area instead of the fixation point at the top of the screen were removed from further analysis (2% of the data). Only sentences with active (SVO) and passive (OVS) structures were scored as correct, but the analyses were limited to active sentences. Thus all trials with passive descriptions, incomplete descriptions (e.g., not naming or incorrectly naming one of the characters), or corrections during the description were excluded from further analysis (*Literal*: 6.3%; *Associative*: 9.3%; *No Mention*: 7.2%). Speech onset latencies shorter or longer than two standard deviations were also removed from the analysis (0.9% of the data). This left 672 trials for analysis.

Analyses were first carried out on speech onsets after applying a log transformation to remove the intrinsic positive skew of RT distributions (Baayen et al., 2008). Mixed-effects models were run with participants and items as random effects, Condition (*Literal. Associative*, and *No Mention*) as a fixed effect, and by-subject and by-item random slopes for Condition. The three conditions were compared with two contrasts using treatment coding. The first contrast compared the *No Mention* condition against the *Literal* condition. The second contrast compared the *No Mention* condition against the *Associative* condition. Both contrasts thus assessed how planning a sentence in response to a story that mentions one of the event characters changed response latencies relative to the *No Mention* condition. Finally, separate analyses were run with new contrasts to compare speech onsets in the *Literal* and *Associative* conditions.

The time-course of sentence formulation in the three conditions was compared with by-participant (β*_1_*) and by-item (β*_2_*) quasi-logistic regression analyses performed on agent-directed fixations (Barr, 2008). We selected two time windows (0–400 ms and 400–1400 ms) for analysis, consistent with earlier analyses of eye movement patterns during spontaneous production of similar descriptions. Fixations were aggregated into a series of time bins of 200 ms for each participant and each item in each condition^1^. The dependent variable in each time bin was an empirical logit indexing the likelihood of speakers fixating the agent out of the total number of fixations observed in that time bin. Time and Condition were entered as fixed effects into all models. All models also included random by-participant and by-item intercepts and slopes for the Time and Condition variables. Main effects in these analyses indicate differences across conditions in the first bin of each window. Interactions with Time show how fixation pattern changed over the remaining bins in that time window.

Similarly to the speech onset analyses, fixations in the three conditions were compared with two contrasts (the *No Mention* condition against the *Literal* and *Associative* conditions separately), and the *Literal* and *Associative* conditions were then compared in a separate analysis.

### Results

#### Speech Onsets

No significant differences were found in speech onset latencies between the *Literal. Associative*, and *No Mention* conditions (all *t*s < 1.5; see **Table 1** for means).

**Table 1:** Mean response latencies in ms (with standard deviation) per condition in Experiment 1 (manipulating Agent Accessibility) and in Experiment 2 (manipulating Patient Accessibility).

	*Literal* Mention	*Associative* Mention	*No Mention*
Experiment 1	1841 (489)	1874 (551)	1866 (496)
Experiment 2	1882 (628)	1944 (519)	1895 (508)


#### Time-Course of Sentence Formulation

**FIGURE 2:**
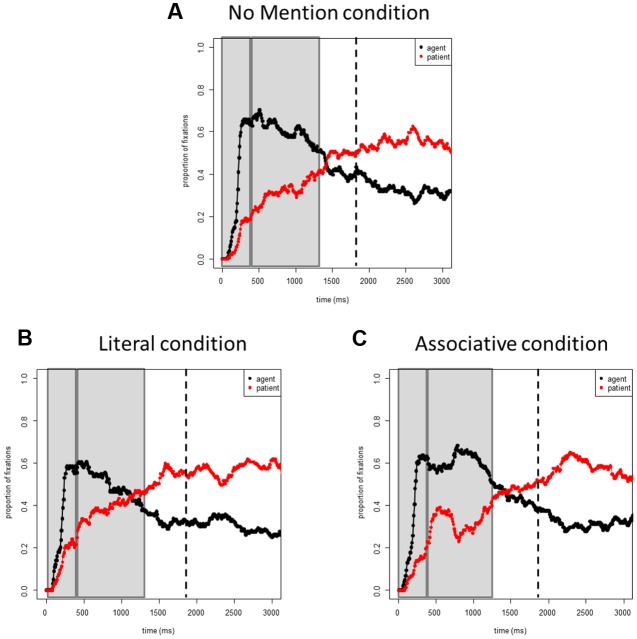
**Experiment 1 (Agent Accessibility).** Proportions of fixations to the agents and patients in the target event pictures: **(A)** *No Mention* condition, **(B)** *Literal* condition, **(C)** *Associative* condition. Time 0 corresponds to picture onset in all figures. Dashed lines represent speech onset. Areas selected by rectangles depict the two time windows (0–400 and 400–1400 ms) used in the analyses.

**Figures 2A–C** shows the proportions of fixations to the agent and patients in target pictures across the three conditions. **Figure 4A** plots the proportions of fixations to the agent in the target pictures for each condition.

##### 0–400 ms

In all conditions, speakers rapidly directed their gaze to the agent in the picture (main effect of Time: β_1_ = 6.09, β_2_ = 6.07, both *t*s > 19), suggesting linearly incremental planning. Neither the main effects of Condition nor the interactions with Condition in this time window were significant (all *t*s < 1).

##### 400–1400 ms

After 400 ms, there were fewer fixations to the agent in the *Literal* condition than in the *No Mention* condition (β_1_ = 0.42, β_2_ = 0.35, both *t*s > 2.9). Speakers were also somewhat less likely to fixate the agent in the *Associative* condition than in the *No Mention* condition. However, this effect reached significance only in the by-subject analysis (β_1_ = -0.26, *t* < -1.99; β_2_ = -0.14, *t* < -1.3), which is likely due only to the small and transient difference between conditions observed between 400 and 800 ms. Comparing the *Literal* and *Associative* conditions against one another in a separate analysis showed that there were fewer looks to the agent in the *Literal* condition than in the *Associative* condition (β_1_ = -0.39, β_2_ = -0.23, both *t*s < -2.4). Including an interaction between Time and Condition did not improve model fits relative to the additive models (χ^2^ = 0.89, *p* > 0.05).

### Discussion

In all three conditions, speakers fixated characters in the order of mention: first the agent (e.g., *frog*) and then the patient (e.g., *fly*). Gaze shifts to the patient occurred prior to speech onset. This pattern generally replicates earlier findings (e.g., Griffin and Bock, 2000), but more specifically, the sharp rise of gaze fixation proportions to the agent within the 0–400 ms window is more consistent with linearly than structurally incremental planning (Gleitman et al., 2007; Kuchinsky, 2009).

The results also show that during the earliest stages of formulation (0–400 ms), character accessibility did not exert an influence on the allocation of attention to the two event characters. This is in line with previous findings showing that information status (i.e., whether information is new and therefore focused or not) does not affect early planning strategies (e.g., Ganushchak et al., 2014). In the present study, eye movements were affected by character accessibility only in later time windows (i.e., after 400 ms). We expected speakers to have a strong preference to fixate the *new*, and thus not accessible, character in the event with priority (*No Mention* condition) and for the length of fixations on this character to vary depending on the discourse context in the remaining conditions (*Literal* and *Associative* conditions). As predicted, there were fewer agent-directed fixations in the *Literal* condition, where the agent was *fully given* than in the *No Mention* condition in the second time window (400–1400 ms). The *Associative* condition, which in terms of character accessibility lies in between the *Literal* and *No Mention* condition, showed little difference from the *No Mention* condition.

Thus, how does accessibility influence the time-course of formulation? In all three conditions, the planning of the message and sentence occurred in a highly incremental manner, consistent with linear incrementality. As we discussed earlier, when speakers first see a picture of an event, they can choose to either “explore” the entire picture to encode its gist (hierarchical planning) or immediately focus on one character in the picture and start building a sentence with that character in subject position (linear planning). Hierarchical planning begins with a convergence of fixations to agents and patients immediately after picture onset (the 0–400 ms time window; Griffin and Bock, 2000 and Konopka and Meyer, 2014, under some conditions). Linear planning begins with a clear divergence of fixations to agents and patients within 200 ms of picture onset and is followed by an extended window with subject fixations (Gleitman et al., 2007). As shown in multiple studies since Meyer et al. (1998), speakers carry out linguistic encoding during such extended fixations to a particular entity (also see Griffin, 2001, 2004; Meyer and Lethaus, 2004; and many others). Our results suggest highly linear incremental planning: speakers fixated the subject character with priority very quickly after picture onset, which implies that they were encoding primarily this character conceptually and linguistically, while the large shift of gaze to the object character around speech onset marks the much later onset of conceptual and linguistic encoding for this character.

Effects of accessibility were observed only at the level of linguistic encoding. In the *Literal* condition, there was a significant reduction of fixations to the agent from 400 ms onward, compared to that in the *No Mention* and *Associative* conditions. This reduction can be explained by the fact that in the *Literal* condition, the agent was explicitly mentioned in the preceding context, thereby reducing the costs of retrieving the lexical and phonological form of this character name (e.g., Ganushchak et al., 2014; Konopka, 2014). In the *Associative* condition, speakers also showed a small and transient reduction in fixations to the agent compared to the *No Mention* condition. This reduction was weaker than in the *Literal* condition, presumably due to the fact that the agent was only activated conceptually by the preceding discourse and the speaker still needed to encode a character name at the lexical and phonological level. This pattern is also in line with the small delay in the onset speech latency in this condition: although this effect did not reach significance, the general trend is that speakers started speaking later in the *Associative* condition than in the other two conditions. This is consistent with the previous literature showing that linguistically given referents had a substantially larger effect on production (e.g., larger acoustic changes) than non-linguistically given referents (e.g., Baumann and Hadelich, 2003; Kahn and Arnold, 2012). To conclude, our results did show that sentence planning in a discourse context can be affected by the degree of character accessibility. On the one hand, there is a clear difference between the *Literal* condition and the *No Mention* condition, which shows that linguistically mentioned referents can facilitate planning of the utterance. On the other hand, the difference between the *Associative* and *No Mention* condition was small, suggesting that the referents that are not linguistically mentioned but are only conceptually accessible (*Associative* condition) are planned in a similar way to referents that are neither linguistically given nor conceptually accessible (*No Mention* condition). This would suggest that what matters more during linguistic encoding is whether a referent is linguistically given or not (i.e., conceptual accessibility without linguistic mention is not sufficient to produce a difference in the time-course of planning).

In Experiment 2, we examine whether utterance planning is affected by the different levels of accessibility of the patient as well.

## Experiment 2: Accessibility of the Patient

### Method

#### Participants

Thirty-one new native Dutch speakers (28 women) participated in the experiment (mean age: 20 years; *SD* = 1.9 years) from the same participant pool as in Experiment 1. Due to a technical problem, data of one participant was excluded from the analysis.

#### Materials, Design, Procedure, and Data Analysis

The materials were similar to the ones in Experiment 1, with the difference that the patient rather than the agent was literally mentioned (the *Literal* condition) or primed (the *Associative* condition) in the stories that preceded the pictures. The *No Mention* condition was identical in both experiments (see below for examples of the stories).

(1) *No Mention* condition: The story did not mention the words describing the target character or include associatively related words.
  *David gaat met zijn vader vissen. Ze gebruiken restjes van het avondeten als aas*. (David is going fishing with his father. They use leftovers from dinner as bait.)
(2) *Literal* condition: The patient was explicitly mentioned.
  *David vist regelmatig en weet dus ook het een en ander over vissen. Hij gebruikt een kleine vlieg als aas.* (David goes fishing often and knows a lot about fishing. He uses a small fly as bait.)
(3) *Associative* condition: The story primed activation of the intended target word. Similar to Experiment 1, stories with the highest agreement on the intended target word were chosen from the pilot study for the main experiment (agreement > 80%).
  *In de zomer zijn er meer insecten in de natuur. Sommige zijn zwart en hebben vleugels.* (During summer, there are a lot of insects in nature. Some are black with wings.)

The design, procedure and analyses were identical to Experiment 1. The target pictures remained on the screen for an average of 4467 ms (*SD* = 1263 ms). Target trials on which erroneous responses were given were removed from further analysis (*Literal*: 7.7%; *Associative*: 8.8%; *No Mention*: 7.1 %). In addition, 1.5% of trials were removed because the first fixation was within the agent or patient interest area instead of the fixation point. This left 696 trials for analysis.

### Results

#### Speech Onsets

There were no significant differences in speech onset latencies between the *Literal. Associative*, and *No Mention* conditions (all *t*s < 1.7; see **Table 1** for means).

#### Time-Course of Sentence Formulation

**FIGURE 3:**
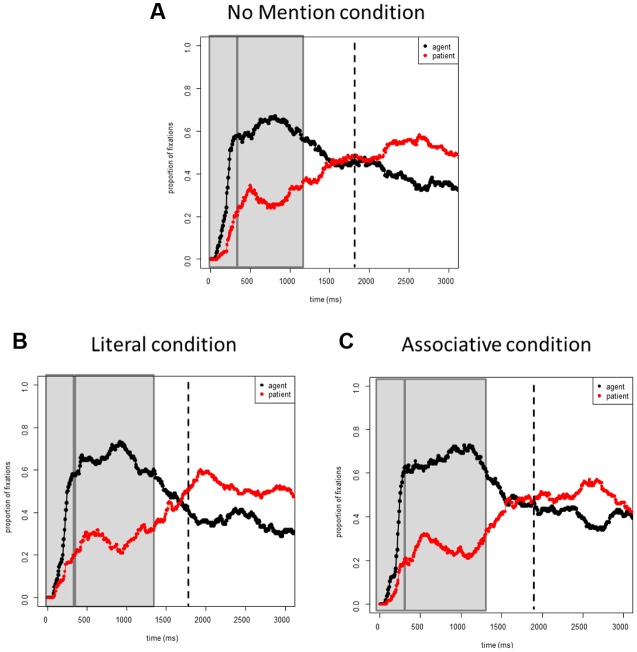
**Experiment 2 (Patient Accessibility).** Proportions of fixations to the agents and patients in the target event pictures: **(A)** the *No Mention* condition; **(B)** the *Literal* condition; **(C)** the *Associative* condition.

**FIGURE 4:**
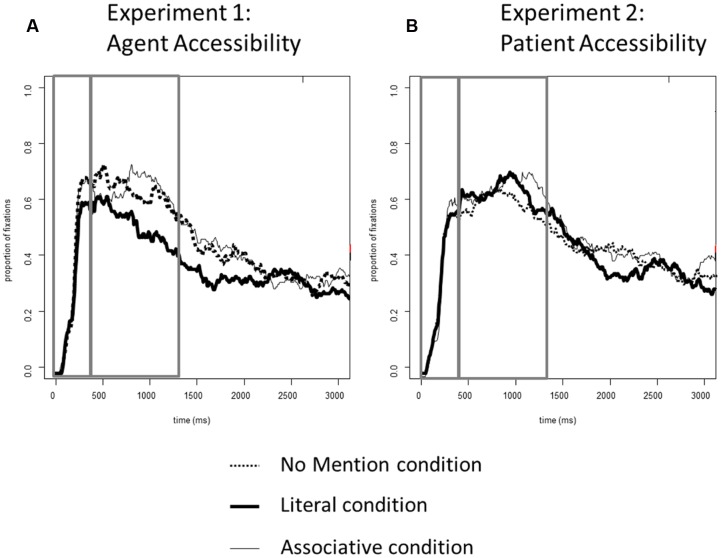
**Proportions of fixations to the agents in target event pictures across all conditions:** **(A)** Experiment 1 (Agent Accessibility); **(B)** Experiment 2 (Patient Accessibility).

**Figures 3A–C** shows the proportions of fixations to the agent and patients in target pictures across conditions. **Figure 4B** plots the proportions of fixations only to the agent in the target pictures for each condition.

##### 0–400 ms

No main effects or interactions were significant (all *t*s < 1).

##### 400–1400 ms

No main effects or interactions were significant (all *t*s < 1.1).

### Discussion

In Experiment 2, participants again looked at the event characters in the order of mention. However, contrary to the findings of Experiment 1, the accessibility of the patient, manipulated in the preceding discourse context, did not affect early (0–400 ms) or late (400–1400 ms) gaze patterns.

What might explain the lack of effects of Condition (i.e., patient accessibility) on formulation in the current study? One possibility is that effects of accessibility of the patient may be observed only at the level of structure choice. There is ample evidence that speakers have a strong preference to begin their sentences with accessible characters. In the current experiment, patients were more accessible in the *Literal* and *Associative* conditions and hence should have been more likely to become sentence subjects than in the *No Mention* condition (where the patient was less accessible as it was not given in the discourse). Speakers did indeed produce more sentences with the intended patient in subject position in the *Literal* (2.2%) and *Associative* (2.5%) conditions than in the *No Mention condition* (1%), but production of such sentences was too infrequent to allow statistical analysis.

At the level of sentence formulation, a possible reason for the lack of an effect of Condition is that speakers were planning the utterance in a very linearly incremental fashion. As in Experiment 1, they started formulation by encoding only the agent with priority. Thus, by the time they started to encode the patient (possibly after starting to speak), the degree of accessibility of the patient was of little consequence for the planning of the sentence. At present, it is also difficult to identify differences in patterns of fixations to a character produced relatively late in a sentence due to increasing variability in the data with each time bin. These results differ from earlier studies which did show that explicit mention of a patient in a question preceding the target pictures (*Who is stopping the truck?*) reduced the costs of retrieving its name as well as the length of fixations on this character (e.g., Konopka, 2013; Ganushchak et al., 2014). Thus, a second reason for the lack of a patient accessibility effect could be the nature of the discourse context itself. Ganushchak et al. (2014) asked wh-questions before showing speakers events to describe. The questions set up a context that was clearly related to either the agent or the patient of the event. Thus, for the speakers, it was clear what information mentioned in the preceding context *should* be part of their response. In the present study, however, participants heard short stories that did not provide unambiguous and consistent cues as to which concepts from the preceding discourse would be part of the upcoming, to-be-produced sentence. In addition, speakers might have tried to suppress activation of information related to the patient from the preceding context in order to start planning sentences beginning with the agent (i.e., active, SVO sentences). This is supported by the somewhat longer speech onset latencies in Experiment 2 compared to Experiment 1^2^.

Finally, in our study, items were strongly biased toward events that elicit SVO sentences. As mentioned earlier, 100% of our target pictures had human/animate agents, and only 52% had human/animate patients. Speakers tend to produce SVO sentences when the agent is human/animate and when the agent and patient are of similar animacy, and are thus reluctant to use non-canonical structures (e.g., Bock et al., 1992; Ferreira, 1994; Branigan et al., 2008). Importantly, however, even in the absence of an effect of context on sentence structure, there are differences in the time-course of formulation (see below for a joint analysis of the data in Experiments 1 and 2). Further research is needed to examine what factors modulate the effect of accessibility of characters that are not produced in sentence-initial position on sentence planning.

Overall, our results show that the conceptual and linguistic planning of a patient occurs possibly as late as after speech onset. This is consistent with the prediction that, since only the first phrase of the sentence is planned conceptually and linguistically prior to articulation, only the availability of lexical items produced in that initial phrase (i.e., the agent name) influences planning at this stage. Allum and Wheeldon (2007, 2009) showed a similar effect in experiments where participants previewed pictures of objects (e.g., a *flower*) that they were to use in a subsequent sentence (e.g., *The flower above the dog is red; The dog above the flower is red*). In these studies, speech onsets were shorter only when the sentence-initial picture was previewed but not when participants previewed a picture whose name was produced in the second phrase. Similarly, Griffin (2001) showed that, when producing highly schematic sentences like *The A and the B are above the C*, speech onsets and gaze durations on the first object were unaffected by properties of the second object. Finally, Schriefers et al. (1998) showed that verb retrieval occurred before sentence onset only when the verb was produced in sentence-initial position. Such findings suggest that the *linguistic* planning of content words in simple sentences indeed occurs in a highly linearly incremental way in sentences with repeated syntactic structures.

### Experiment 1 vs. Experiment 2

Finally, we assessed differences between the accessibility of agents and patients on utterance formulation in a joint analysis for Experiments 1 and 2. In these analyses, the fixed factor Experiment was added into all the models and the analyses were carried out as described above (i.e., Time and Condition were entered as fixed effects into all models; all models also included random by-participant and by-item intercepts and slopes for the Time and Condition variables.). The log-likelihood ratio test (χ^2^) was used to compare model fits in interactive and additive models, and thus test whether interactions with the Experiment variable significantly improved model fit. A reliable difference in this comparison indicates a better fit for the interactive model than the additive model.

#### 0–400 ms

There was no difference between Experiment 1 and Experiment 2 (all interactions with Experiment: *t*s < 1). In addition, adding the Experiment variable into the model did not improve model fit relative to a model without this variable (χ^2^ = 1).

#### 400–1400 ms

For all of the models, adding Experiment as a fixed factor significantly improved model fit (χ^2^ < 0.01), and there was a significant interaction between Experiment and Time for all contrasts (*Literal* vs. *Associative*: β_1_ = -0.44, β_2_ = -0.34, both *t*s < -3.0; *Literal* vs. *No Mention*: β_1_ = -0.46, β_2_ = -0.37, both *t*s < -3.0; *Associative* vs. *No Mention*: β_1_ = -0.48, β_2_ = -0.42, both *t*s < -3.0). Overall, there were more looks to the agent in Experiment 2 (manipulating accessibility of the patient) than in the Experiment 1 (manipulating accessibility of the agent). Additionally, the comparison between the *Literal* and *No Mention* conditions showed a significant interaction between Experiment and Condition (β_1_ = 0.53, β_2_ = 0.45, both *t*s < -3.3), as there were fewer looks to the agent in the *Literal* condition in Experiment 1 than in Experiment 2. This confirms that changes in agent-directed fixations were driven by the degree of accessibility of the referent.

## General Discussion

In two experiments, we compared the time-course of formulation for sentences produced when two event characters (agents and patients) varied in terms of their contextual accessibility. The discourse context either provided no information about the target event (*No Mention* condition), specifically mentioned one of the event characters (*Literal* Agent and Patient conditions), or was associatively related to one of the characters (*Associative* Agent and Patient conditions).

The results showed that character accessibility did not influence the distribution of attention to the two event characters immediately after picture onset (0–400 ms) but only later, after 400 ms. Thus, the discourse manipulation did not affect *when* speakers began encoding a given character, but rather affected the length of time needed to encode that character. Overall, only the accessibility of the agent (i.e., the sentence-initial character) and not the accessibility of the patient (i.e., the sentence-final character) affected gaze patterns, suggesting linearly rather than hierarchically incremental planning.

This suggests that information activated by the discourse context (conceptual, lexical, and phonological information in the *Literal* condition; conceptual information in the *Associative* condition) can facilitate planning but only when it is relevant for the initial word/phrase of the sentence. Our results show that the planning of the patient occurred after planning of the agent, likely around speech onset, and thus was not part of the initial preverbal message generated at picture onset. We conclude that when the discourse activates information that is relevant for phrases further downstream (i.e., after the sentence-initial word/phrase), it is less beneficial for planning since it falls outside the scope of the first planning window. This is in line with previous findings that showed that effects of lexical activation are limited only to the production of a sentence-initial word (e.g., Meyer, 1996; Wheeldon and Lahiri, 1997; Griffin, 2001; Costa et al., 2006; Allum and Wheeldon, 2007, 2009). The exact timing of the planning of the patient relative to speech onset may vary with the ease of encoding the agent: rapid encoding of the agent likely allows an earlier shift of gaze and attention to the patient (as in Experiment 1), while slower encoding of the agent likely results in a delayed shift of gaze and attention to the patient (as in Experiment 2). The difference in timing between the shift of gaze to the patient and speech onset across experiments may suggest a change in late planning strategies that deserves further attention in future work.

Finally, the discourse context in the present study was not intended to help participants understand the gist of the depicted events, although we cannot exclude the possibility that it provided conceptual information that facilitated gist encoding. Furthermore, we cannot exclude the possibility that the fact that the structure of to-be-produced sentences was repeated in Experiments 1 and 2 led to more linear planning than would have been the case when structure was not repeated (e.g., Konopka and Kuchinsky, 2015). For instance, Konopka and Kuchinsky (2015) used conceptual and structural primes before asking participants to describe target events (similar to the *Associative* and *No Mention* conditions in the present study), and found that both types of primes jointly affected the formulation of target descriptions on both the message and sentence levels, albeit with much stronger effects at the sentence level. It is possible that event gist was also primed by the discourse context in our study but that it did not influence formulation due to repeated production of one sentence type (SVO).

A final theoretical question concerns the difference between *accessibility* and *givenness*. Hypothetically, one could reduce the effect of discourse accessibility in these experiments to mere lexical priming and thus attribute the observed differences between conditions to differences in levels of *word* activation, or linguistic *givenness*. Givenness in the existing linguistic literature has often been operationalized as a binary classification of a referent being either *given* or *new* (but see Chafe, 1976; Ariel, 1990; Gundel et al., 1993), and a referent is defined as being “given” in a particular context specifically if it has been linguistically mentioned. Givenness has been shown to influence, for example, the form of referential expressions and their acoustic/phonetic realization: referential expressions used for known referents tend to be shorter and have different intonation contours (e.g., Brown, 1983; Pierrehumbert and Hirschberg, 1990; Baumann, 2006; Chen, 2010; see also Ladd, 2008; Baumann and Riester, 2012, for further discussion and references). Listeners have also been shown to be sensitive to the various prosodic cues that speakers employ to signal different levels of givenness (Baumann and Grice, 2006).

In our study, the givenness of a referent was operationalized as *accessibility* at two levels of that referent’s representation, namely accessibility at the conceptual level (in the *Associative* condition) and accessibility at the linguistic level (in the *Literal* condition). Our results show that different levels of accessibility affected planning in different ways, and while more research is needed to identify the mechanism(s) behind effects of lexical primes and discourse primes on sentence formulation, we propose that givenness can also be viewed beyond the given vs. not given dichotomy. Instead, it may, and probably should, be viewed as a variable that, together with *accessibility*, expresses differences in the activation of a referent during conceptual and linguistic planning.

## Conclusion

Our results show that sentence planning can be affected by the relative accessibility of event characters, such that in situations where the discourse context does not provide clear cues about the sequential position of these concepts in a potential utterance, character accessibility impacts sentence formulation only when the accessible character is mentioned in sentence-initial position.

## Ethics Statement

The study was approved by the ethical committee board at Leiden University. All participants were adults. At the beginning of the experimental session, they read the instructions describing the experiment and were asked whether they are happy to proceed. The instruction describes the task and structure of the experiment. The instructions stress that the participant is free to leave the experiment at any time without providing any explanation to the experimenter. If the participant is happy to proceed, they sign the consent form and the experiment commences. If a person decides not to participate, the experimenter will reassure him/her that this is entirely acceptable and thank him/her for his/her interest.

## Author Contributions

LG: Substantial contributions to the conception or design of the work; the acquisition, analysis, and interpretation of data for the work; drafting the work and revising it critically. AK: Substantial contributions to the design of the work; interpretation of data for the work; critically revising the manuscript. YC: Substantial contributions to the conception or design of the work; interpretation of data for the work; critically revising the manuscript. LG, AK, and YC: Final approval of the version to be published; agreement to be accountable for all aspects of the work in ensuring that questions related to the accuracy or integrity of any part of the work are appropriately investigated and resolved.

## Conflict of Interest Statement

The authors declare that the research was conducted in the absence of any commercial or financial relationships that could be construed as a potential conflict of interest.

## Footnotes

1 We also run analysis with the smaller bins, which resulted in the same results. We chose to report a more conservative analysis with 200 ms bins.

2 Note that similar to our study, in Konopka and Meyer’s (2014) study, participants were also unaware which of the event characters was primed by the preceding context. But they did report that object primes influenced structure choice in planning, which is likely, at least partly, due to the selection of events that were more likely to elicit OVS descriptions.

## References

[B1] Allum P. H. Wheeldon L. R. (2007). Planning scope in spoken sentence production: the role of grammatical units. *J. Exp. Psychol.* 33 791–810. 10.1037/0278-7393.33.4.791 17576154

[B2] Allum P. H. Wheeldon L. R. (2009). Scope of lexical access in spoken sentence production: implications for the conceptual-syntactic interface. *J. Exp. Psychol.* 35 1240–1255. 10.1037/a0016367 19686018

[B3] Ariel M. (1990). *Accessing Noun-Phrase Antecedents.* London: Routledge.

[B4] Baayen R. H. Davidson D. J. Bates D. M. (2008). Mixed-effects modeling with crossed random effects for subjects and items. *J. Mem. Lang.* 59 390–412. 10.1016/j.jml.2007.12.005

[B5] Barr D. J. (2008). Analyzing ‘visual world’ eyetracking data using multilevel logistic regression. *J. Mem. Lang.* 59 457–474. 10.1016/j.jml.2007.09.002

[B6] Baumann S. (2006). *The Intonation of Givenness–Evidence From German (Linguistische Arbeiten 508).* Tübingen: Niemeyer 10.1515/9783110921205

[B7] Baumann S. Grice M. (2006). The intonation of accessibility. *J. Pragmat.* 38 1636–1657. 10.1016/j.pragma.2005.03.017

[B8] Baumann S. Hadelich K. (2003). “Accent type and givenness: an experiment with auditory and visual priming,” in *Proceedings of the 15th ICPhS*, Barcelona, 1811–1814.

[B9] Baumann S. Riester A. (2012). “Referential and lexical givenness: semantic, prosodic and cognitive aspects,” in *Prosody and Meaning*, eds Elordieta G. Prieto P. (Berlin: Mouton De Gruyter), 119–162.

[B10] Bock J. K. (1986). Meaning, sound, and syntax: lexical priming in sentence production. *J. Exp. Psychol.* 12 575–586. 10.1037/0278-7393.12.4.575

[B11] Bock J. K. Irwin D. E. Davidson D. J. J. (2004). “Putting first things first,” in *The Integration of Language, Vision, and Action: Eye Movements and the Visual World*, eds Ferreira F. Henderson M. (New York, NY: Psychology Press), 249–278.

[B12] Bock J. K. Loebell H. Morey R. (1992). From conceptual roles to structural relations: bridging the syntactic cleft. *Psychol. Rev.* 99 150–171. 10.1037/0033-295X.99.1.150 1546115

[B13] Bock J. K. Warren R. K. (1985). Conceptual accessibility and syntactic structure in sentence formulation. *Cognition* 21 47–67. 10.1016/0010-0277(85)90023-X 4075761

[B14] Bock K. Irwin D. E. Davidson D. Levelt W. J. M. (2003). Minding the clock. *J. Mem. Lang.* 48 653–685. 10.1016/S0749-596X(03)00007-X

[B15] Branigan H. P. Feleki E. (1999). “Conceptual accessibility and serial order in Greek language production,” in *Proceedings of the 21st Conference of the Cognitive Science Society*, Vancouver, 96–101.

[B16] Branigan H. P. Pickering M. J. Tanaka M. (2008). Contributions of animacy to grammatical function assignment and word order during production. *Lingua* 118 172–189. 10.1016/j.lingua.2007.02.003

[B17] Brown G. (1983). “Prosodic structure and the given/new distinction,” in *Prosody: Models and Measurements*, eds Cutler A. Ladd R. (Berlin: Springer Verlag), 67–77. 10.1007/978-3-642-69103-4_6

[B18] Chafe W. L. (1976). “Givenness, contrastiveness, definiteness, subjects, topics, and point of view,” in *Subject and Topic*, ed. Li C. N. (New York, NY: Academic Press), 25–56.

[B19] Chen Y. (2010). Post-focus f0 compression–Now you see it, now you don’t. *J. Phon.* 38 517–525. 10.1016/j.wocn.2010.06.004

[B20] Christianson K. Ferreira F. (2005). Conceptual accessibility and sentence production in a free word order language (Odawa). *Cognition* 98 105–135. 10.1016/j.cognition.2004.10.006 16307955

[B21] Clark H. H. Marshall C. R. (1981). “Definite reference and mutual knowledge,” in *Elements of Discourse Understanding*, eds Joshi A. K. Webber B. L. Sag I. S. (New York, NY: Cambridge University Press),k10–63.

[B22] Costa A. Navarrete E. Alario F. X. (2006). Accessing object names when producing complex noun phrases: implications for models of lexical access. *Cognitiva* 18 3–23. 10.1174/021435506775462454

[B23] Ferreira F. (1994). Choice of passive voice is affected by verb type and animacy. *J. Mem. Lang.* 33 715–736. 10.1006/jmla.1994.1034

[B24] Ferreira V. S. Yoshita H. (2003). Given-new ordering effects on the production of scrambled sentences in Japanese. *J. Psycholinguist. Res.* 32 669–692. 10.1023/A:1026146332132 14653013

[B25] Ganushchak L. Y. Konopka A. Chen Y. (2014). What the eyes say about planning of focused referents during sentence formulation: a cross-linguistic investigation. *Front. Psychol.* 5:e1124 10.3389/fpsyg.2014.01124 PMC4183096 25324820

[B26] Gleitman L. January D. Nappa R. Trueswell J. C. (2007). On the give and take between event apprehension and utterance formulation. *J. Mem. Lang.* 57 544–569. 10.1016/j.jml.2007.01.007 18978929 PMC2151743

[B27] Griffin Z. M. (2001). Gaze durations during speech reflect word selection and phonological encoding. *Cognition* 82 B1–B14. 10.1016/S0010-0277(01)00138-X 11672707 PMC5130081

[B28] Griffin Z. M. (2004). The eyes are right when the mouth is wrong. *Psychol. Sci.* 15 814–821. 10.1111/j.0956-7976.2004.00761.x 15563326

[B29] Griffin Z. M. Bock J. K. (2000). What the eyes say about speaking. *Psychol. Sci.* 11 274–279. 10.1111/1467-9280.00255 11273384 PMC5536117

[B30] Gundel J. K. Hedberg N. Zacharski R. (1993). Cognitive status and the form of referring expressions. *Language* 69 274–307. 10.2307/416535

[B31] Gussenhoven C. (2007). “Types of focus in English,” in *Topic and Focus: Cross-Linguistic Perspectives on Meaning and Intonation*, eds Lee C. M. Gordon M. Büring D. (Dordrecht: Springer), 83–100. 10.1007/978-1-4020-4796-1_5

[B32] Kahn J. M. Arnold J. E. (2012). A processing-centered look at the contribution of givenness to durational reduction. *J. Mem. Lang.* 67 311–325. 10.1016/j.jml.2012.07.002

[B33] Kempen G. Harbusch K. (2003). Word order scrambling as a consequence of incremental sentence production. *Trends Linguist. Stud. Monogr.* 152 141–164. 10.1515/9783110919585.141

[B34] Kempen G. Hoenkamp E. (1987). An incremental procedural grammar for sentence formulation. *Cogn. Sci.* 11 201–258. 10.1207/s15516709cog1102_5

[B35] Konopka A. E. (2012). Planning ahead: how recent experience with structures and words changes the scope of linguistic planning. *J. Mem. Lang.* 66 143–162. 10.1016/j.jml.2011.08.003

[B36] Konopka A. E. (2013). “Discourse changes the timecourse of sentence formulation,” in *Poster Presented at the 19th Architectures and Mechanisms for Language Processing Conference*, Marseille.

[B37] Konopka A. E. (2014). “Speaking in context: discourse influences formulation of simple sentences,” in *Poster Presented at the 27th CUNY Human Sentence Processing Conference*, Columbus, OH.

[B38] Konopka A. E. Brown-Schmidt S. (2014). “Message encoding,” in *The Oxford Handbook of Language Production*, eds Goldrick M. Ferreira V. Miozzo M. (Oxford: Oxford University Press), 3–20.

[B39] Konopka A. E. Kuchinsky S. E. (2015). How message similarity shapes the timecourse of sentence formulation. *J. Mem. Lang.* 84 1–23. 10.1016/j.jml.2015.04.003

[B40] Konopka A. E. Meyer A. S. (2014). Priming sentence planning. *Cognit. Psychol.* 73 1–40. 10.1016/j.cogpsych.2014.04.001 24838190

[B41] Krifka M. (2007). “Basic notions of information structure,” in *Interdisciplinary Studies of Information Structure 6*, eds Fery C. Krifka M. (Potsdam: Universitätsverlag).

[B42] Kruijff G.-J. (2001). *A Categorical-Modal Architecture of Informativity: Dependency Grammar Logic and Information Structure.* Ph.D. thesis, Charles University, Prague.

[B43] Kuchinsky S. E. (2009). *From Seeing to Saying: Perceiving, Planning, Producing.* Ph.D. dissertation, University of Illinois at Urbana-Champaign, Champaign, IL.

[B44] Kuchinsky S. E. Bock K. (2010). “From seeing to saying: perceiving, planning, producing,” in *Paper Presented at the 23rd Meeting of the CUNY Human Sentence Processing Conference*, New York, NY.

[B45] Ladd R. (2008). *Intonational Phonology*, 2nd Edn Cambridge: Cambridge University Press 10.1017/CBO9780511808814

[B46] MacWhinney B. Bates E. (1978). Sentential devices for conveying givenness and newness: a cross-cultural developmental study. *J. Verbal Learning Verbal Behav.* 17 539–558. 10.1016/S0022-5371(78)90326-2

[B47] McDonald J. L. Bock K. Kelly M. H. (1993). Word and world order: semantic, phonological, and metrical determinants of serial position. *Cognit. Psychol.* 25 188–230. 10.1006/cogp.1993.1005 8482072

[B48] Meyer A. S. (1996). Lexical access in phrase and sentence production: results from picture–word interference tasks. *J. Mem. Lang.* 35 477–496. 10.1006/jmla.1996.0026

[B49] Meyer A. S. Lethaus F. (2004). “The use of eye tracking in studies of sentence generation,” in *The Interface of Language, Vision, and Action: Eye Movements and the Visual World*, eds Henderson J. M. Ferreira F. (New York, NY: Psychology Press), 191–212.

[B50] Meyer A. S. Sleiderink A. M. Levelt W. J. M. (1998). Viewing and naming objects: eye movements during noun phrase production. *Cognition* 66 B25–B33. 10.1016/S0010-0277(98)00009-2 9677766

[B51] Norcliffe E. Konopka A. E. Brown P. Levinson S. (2015). Word order affects the timecourse of sentence formulation in Tzeltal. *Lang. Cogn. Neurosci.* 30 1187–1208. 10.1080/23273798.2015.1006238

[B52] Pierrehumbert J. Hirschberg J. (1990). “The meaning of intonational contours in the interpretation of discourse,” in *Intentions in Communication*, eds Cohen P. R. Morgan J. Pollack M. E. (Cambridge, MA: MIT Press), 271–311.

[B53] Rochemont M. (2016). *Givenness, Oxford Handbook of Information Structure.* Oxford: Oxford University Press.

[B54] Schriefers H. Teruel E. Meinshausen R. M. (1998). Producing simple sentences: results from picture–word interference experiments. *J. Mem. Lang.* 39 609–632. 10.1006/jmla.1998.2578

[B55] Tanaka M. N. Branigan H. P. McLean J. F. Pickering M. J. (2011). Conceptual influences on word order and voice in sentence production: evidence from Japanese. *J. Mem. Lang.* 65 318–330. 10.1016/j.jml.2011.04.009

[B56] Wheeldon L. R. Lahiri A. (1997). Prosodic units in speech production. *J. Mem. Lang.* 37 356–381. 10.1006/jmla.1997.2517

